# Experimental phasing with vanadium and application to nucleotide-binding membrane proteins

**DOI:** 10.1107/S2052252520012312

**Published:** 2020-10-14

**Authors:** Kamel El Omari, Nada Mohamad, Kiran Bountra, Ramona Duman, Maria Romano, Katja Schlegel, Hok-Sau Kwong, Vitaliy Mykhaylyk, Claus Olesen, Jesper Vuust Moller, Maike Bublitz, Konstantinos Beis, Armin Wagner

**Affiliations:** a Diamond Light Source, Harwell Science and Innovation Campus, Didcot OX11 0DE, United Kingdom; b Research Complex at Harwell, Rutherford Appleton Laboratory, Didcot OX11 0FA, United Kingdom; c Department of Biochemistry, University of Oxford, South Parks Road, Oxford OX1 3QU, United Kingdom; d Department of Life Sciences, Imperial College, London, United Kingdom; e Institute of Biostructures and Bioimaging, National Research Council (IBB–CNR), Via Mezzocannone 16, 80134 Napoli, Italy; f Department of Biomedicine, Aarhus University, Ole Worms Allé 8, DK-8000 Aarhus, Denmark

**Keywords:** experimental phasing, vanadium, membrane proteins

## Abstract

The structure determination of soluble and membrane proteins can be hindered by the crystallographic phase problem, especially in the absence of a suitable homologous structure. Experimental phasing is the method of choice for novel structures; however, it often requires heavy-atom derivatization, which can be difficult and time-consuming. Here, a novel and rapid method to obtain experimental phases for protein structure determination by vanadium phasing is reported. Vanadate is a transition-state mimic of phosphoryl-transfer reactions and it has the advantage of binding specifically to the active site of numerous enzymes catalyzing this reaction. The applicability of vanadium phasing has been validated by determining the structures of three different protein–vanadium complexes, two of which are integral membrane proteins: the rabbit sarcoplasmic reticulum Ca^2+^-ATPase, the antibacterial peptide ATP-binding cassette transporter McjD from *Escherichia coli* and the soluble enzyme RNAse A from *Bos taurus*. Vanadium phasing was successful even at low resolution and despite severe anisotropy in the data. This method is principally applicable to a large number of proteins, representing six of the seven Enzyme Commission classes. It relies exclusively on the specific chemistry of the protein and it does not require any modifications, making it a very powerful addition to the phasing toolkit. In addition to the phasing power of this technique, the protein–vanadium complexes also provide detailed insights into the reaction mechanisms of the studied proteins.

## Abstract 2

Crystal structure determination of membrane proteins can be challenging. Here, the use of vanadium phasing as an alternative experimental phasing method is described.

## 1. Introduction   

Membrane proteins mediate essential processes in all living organisms, including signaling, nutrient uptake, xenobiotic efflux and multidrug resistance. Membrane proteins account for 20–30% of the genome and many of them are important drug targets. The fact that membrane proteins reside within the lipid bilayer makes them notoriously difficult proteins to work with. Besides the difficulties encountered during protein expression, purification and crystallization (Carpenter *et al.*, 2008 ▸), membrane-protein crystals tend to diffract to low resolution and often suffer from high mosaicity and non-isomorphism, rendering structure determination difficult if molecular replacement is not an option. Consequently, amongst the 50 000 distinct proteins in the Protein Data Bank (wwPDBconsortium, 2019 ▸), less than one thousand are membrane proteins (https://blanco.biomol.uci.edu/mpstruc/). Considering that more than 60% of drug targets are membrane proteins (Overington *et al.*, 2006 ▸), there is a strong unmet need to facilitate their structure determination.

Currently, molecular replacement is the method of choice for crystal structure determination. However, this method is not suitable for *de novo* structure determination and can introduce model bias, especially for crystals that diffract to low resolution. On the other hand, experimental phasing does not need a protein model and is bias-free, but entails targeting atoms with anomalous scattering properties that can be exploited. Single-wavelength anomalous diffraction (SAD) is currently the main experimental phasing technique (Rose & Wang, 2016 ▸) that only requires data collected at a single X-ray wavelength, thus differing from multiple-wavelength anomalous dispersion (MAD), where at least two data sets at different wavelengths are measured from a single crystal or from multiple isomorphous crystals. In the case of SAD, the phase-ambiguity problem is resolved by density-modification methods (Hendrickson, 2014 ▸; Wang, 1985 ▸).

Numerous atoms can be used as anomalous scatterers for experimental phasing, ranging from the commonly used selenium to transition metals such as mercury, gold and platinum (heavy atoms) or even lighter atoms such as sulfur, potassium and calcium that occur naturally in proteins and are used for native phasing. Selenium can be directly incorporated into proteins during their synthesis, while other scatterers are normally soaked or co-crystallized for incorporation (Dauter & Dauter, 2017 ▸). Protein labeling is not always possible or can decrease protein-expression yields, whereas heavy-atom derivatization is essentially a trial-and-error method that can fail because of nonspecific binding or loss of isomorphism. New molecules that can be used for experimental phasing and present more specificity for proteins have been investigated; amongst many others, the magic triangle (Beck *et al.*, 2008 ▸) and iodo-detergents (Nakane *et al.*, 2016 ▸) are good examples.

Vanadium, in its tetrahedral orthovanadate form (VO_4_^3−^) or as the planar metavanadate (VO_3_^−^), is a well known transition-state mimic of phosphoryl-transfer reactions and hence is a potent inhibitor of many phosphoryl-transfer enzymes (Davies & Hol, 2004 ▸). Compared with other transition-state mimics such as aluminium- or beryllium fluoride, vanadate also has the benefit of readily forming covalent bonds with a variety of ligands. Indeed, vanadate can form covalent bonds with serine and tyrosine residues in catalytic sites (Davies *et al.*, 2006 ▸; Holtz *et al.*, 1999 ▸) or even a cyclic complex with the ribose of a nucleotide (Ladner *et al.*, 1997 ▸). It is also possible to mimic the transition state of topoisomerase IB, where a covalent DNA–vanadate–protein complex is formed (Davies *et al.*, 2006 ▸). Hence, it is not surprising that vanadium is found in a variety of protein complexes in the PDB (Davies & Hol, 2004 ▸): phosphodiesterases, ATPases, phosphotransferases, phosphatases, sulfotransferases, ribonucleases, mutases, kinases and chloroperoxidases (Davies & Hol, 2004 ▸). Many members of these enzyme families are membrane proteins, which often form poorly diffracting crystals and pose a challenge for low-resolution phasing.

Knowing that vanadate binds with high specificity to several enzyme classes containing membrane proteins, we investigated its anomalous scattering properties in order to assess its use as a novel protein-specific anomalous scatterer for experimental phasing. In contrast to the absorption spectra of other elements, vanadium, and in particular vanadate, does not show a white-line feature with a very distinct peak (Supplementary Fig. S1). For vanadate, a pre-edge peak is typically followed by the absorption edge and its X-ray absorption near-edge structure (XANES) features (Levina *et al.*, 2014 ▸). Hence, the strategy was to perform a SAD experiment at an energy above the theoretical absorption edge to exploit the anomalous term *f*′′ of approximately 4 electrons. In order to test whether vanadium can indeed be used for experimental phasing, we collected long-wavelength X-ray diffraction data from three known protein–vanadate complexes for V-SAD phasing: two membrane-transport proteins, the antibacterial peptide ATP-binding cassette (ABC) transporter McjD from *E. coli* (Choudhury *et al.*, 2014 ▸) and the rabbit sarcoplasmic reticulum Ca^2+^-ATPase (SERCA; Clausen *et al.*, 2016 ▸), and a soluble enzyme, bovine pancreatic ribonuclease A (RNAseA; Ladner *et al.*, 1997 ▸). McjD is a membrane protein of ∼66 kDa belonging to the ABC transporter superfamily which binds and hydrolyses ATP for the export of various substrates. The protein exists as a dimer and provides bacterial cells with self-immunity against the antibacterial peptide MccJ25 (Choudhury *et al.*, 2014 ▸). Previous determination of the McjD–ADP–VO_4_ structure (Bountra *et al.*, 2017 ▸) showed McjD in a post-ATP-hydrolysis state by the mimicry of the transition state by vanadate. SERCA is a larger membrane protein of 110 kDa that belongs to the family of P-type ATPases (Bublitz *et al.*, 2011 ▸). Its function during muscle relaxation is to pump Ca^2+^ from the cytosol to the sarcoplasmic reticulum at the expense of ATP hydrolysis. The structure of SERCA–VO_3_ has also previously been reported (Clausen *et al.*, 2016 ▸). In contrast to the tetrahedral VO_4_ form bound to McjD, the vanadate species bound to SERCA has been modeled in its trigonal planar VO_3_ state, mimicking the pentacovalent transition state of dephosphorylation. To further assess the suitability of vanadate as a phasing tool in protein–nucleotide complexes rather than in ATPases, we investigated its ability to phase RNAse A, a small protein of ∼14 kDa that catalyzes the hydrolysis of phosphate–ester linkages in single-stranded RNA. RNAse A binds vanadate in a very different manner compared with McjD and SERCA. The vanadate is covalently bound to the ribose moiety of uridine, forming a pentacoordinate phosphorane analog (uridine–vanadate, UV; Ladner *et al.*, 1997 ▸).

This study demonstrates the successful use of vanadium SAD phasing to determine three protein–vanadate complex structures, two of which are membrane-protein representatives from the ABC transporter and P-type ATPase families, the structural determination of which had previously been dependent on molecular replacement. To our knowledge, this is the first time that vanadium phasing has been reported.

## 2. Materials and methods   

### 2.1. Preparation of vanadate   

Orthovanadate-enriched stock solutions were prepared following established protocols (Ko *et al.*, 1997 ▸; Varga *et al.*, 1985 ▸). 60 m*M* orthovanadate stock solution was prepared by dissolving Na_3_VO_4_ in water and adjusting the pH to 10.0 with HCl. The solution was boiled for 2 min, cooled on ice and the pH was readjusted to 10.0 with NaOH. The procedure was repeated twice, whereupon the pH remained stable at 10.0. The orthovanadate concentration was measured spectrophotometrically at 265 nm using an extinction coefficient of 2925 *M* ^−1^ cm^−1^. A 20 m*M* decavanadate stock solution was prepared by dilution of the orthovanadate stock solution with water (1:2 dilution factor) and the pH was adjusted to 2.0 with 5 *N* HCl. The solution was equilibrated for a few hours at 4°C, followed by readjustment of the pH to 6.5 with NaOH. The orthovanadate and decavanadate stock solutions were stored at −80 and 4°C, respectively.

### 2.2. Protein production and crystallization   

McjD was purified in 20 m*M* Tris pH 7.8, 150 m*M* NaCl, 0.03% dodecyl maltopyranoside as described previously, without modifications (Bountra *et al.*, 2017 ▸; Choudhury *et al.*, 2014 ▸). The McjD–ADP–VO_4_ complex was obtained by incubating 15 mg ml^−1^ McjD with 2 m*M* ATP, 2 m*M* sodium orthovanadate and 5 m*M* MgCl_2_ for 1 h at room temperature. Crystals were grown at 293 K using the vapor-diffusion method in a condition consisting of 10% PEG 4000, 100 m*M* ammonium sulfate, 100 m*M* HEPES pH 7.5, 22% glycerol. After four days, the crystals were directly flashed-cooled in liquid nitrogen.

SERCA (isoform 1a) purification took place as described previously (Clausen *et al.*, 2016 ▸; Winther *et al.*, 2013 ▸). The protein was purified from sarcoplasmic reticulum vesicles isolated from rabbit hind-leg skeletal muscle. Low concentrations of deoxycholate were used to further extract and purify membranes containing SERCA according to previous procedures (Andersen *et al.*, 1985 ▸). The membrane preparation was cleaned by centrifugation at 130 000*g* for 35 min at 4°C in 100 m*M* MOPS–Tris pH 6.8, 80 m*M* KCl. The pellet was resuspended in centrifugation buffer plus 20%(*v*/*v*) glycerol, 1.5 m*M* EGTA, 0.25–0.4 m*M* MgCl_2_ and supplemented with 1 m*M* orthovanadate-enriched solution, followed by incubation for 1–2 h on ice. TNP-ATP was added to give a final concentration of 0.4 m*M*, followed by a 15–30 min incubation on ice. Finally, the protein was solubilized by the addition of octaethyleneglycol dodecylether [detergent:protein ratio of 1.5:1(*w*:*w*)], incubated for 15–30 min on ice and centrifuged at 130 000*g* for 35 min at 4°C. The supernatant, with a protein content of ∼10 mg ml^−1^, was used directly in crystallization trials using the vapor-diffusion technique in hanging crystallization drops (1–5 µl) that were manually pipetted onto siliconized glass cover slides (Hampton Research) sealed to the reservoir (400 µl of the buffer) with microscopy immersion oil. Drops containing protein solution and crystallization buffer were mixed in a 1:1 ratio. The crystals grew in 19% PEG 2000 MME, 11% glycerol, 0.1 *M* MgCl_2_, 6% 1-butanol and were directly flash-cooled in liquid nitrogen.

Bovine pancreatic RNase A powder from Sigma was dissolved in water at a concentration of 18 mg ml^−1^. Crystals of RNase A were grown at 289 K using hanging-drop vapor diffusion, as described previously (Leonidas *et al.*, 1997 ▸). The uridine vanadate–RNase A complex was obtained by soaking RNase A crystals in 20 m*M* sodium citrate pH 5.5, 28% PEG 4000 with 20 m*M* uridine–orthovanadate (1:1 ratio) for at least 16 h prior to data collection and the crystals were cryoprotected by transfer into crystallization solution containing 20%(*v*/*v*) 2-methyl-2,3-pentanediol.

### 2.3. Data-collection, structure solution and refinement   

All diffraction data were collected on a PILATUS 12M detector at ∼60 K on I23 (Wagner *et al.*, 2016 ▸), the long-wavelength beamline at Diamond Light Source (DLS). The wavelength was chosen to be close to the V *K* edge (λ = 2.2604 Å).

For the McjD–ADP–VO_4_ complex, 360° of data were recorded using the inverse-beam method (20° wedges). McjD crystals typically suffer from anisotropy (Choudhury *et al.*, 2014 ▸), and hence *autoPROC* (Vonrhein *et al.*, 2011 ▸) and *STARANISO* (Tickle *et al.*, 2018 ▸) were used for processing and anisotropy correction (Table 1 ▸). Vanadium sites were found with *SHELXD* (Sheldrick, 2010 ▸) from the *HKL*2*MAP* suite (Pape & Schneider, 2004 ▸), looking for two sites with a resolution cutoff of 6 Å over 500 tries. The vanadium sites were used as input for *phenix.autosol* (Liebschner *et al.*, 2019 ▸). The initial experimental maps clearly showed the shape of McjD and the transmembrane helices; however, the side-chain positions were difficult to distinguish. The experimental maps were used as a search model in *Phaser* (McCoy *et al.*, 2007 ▸) against a higher resolution data set (2.7 Å) with a different space group (*P*2_1_2_1_2_1_; Choudhury *et al.*, 2014 ▸; Table 1 ▸). Model building was undertaken in *Buccaneer* (Cowtan, 2006 ▸) and *Coot* (Emsley *et al.*, 2010 ▸). Refinement of McjD was achieved with *phenix.refine* (Liebschner *et al.*, 2019 ▸).

From a single crystal of SERCA, 12 data sets of 360° each were collected with different κ and φ angles. The data sets were processed using *XDS*, merged with *XSCALE* (Kabsch, 2010 ▸) and converted with *AIMLESS* (Evans & Murshudov, 2013 ▸). An initial attempt to solve the vanadium substructure was successfully carried out with the *HKL*2*MAP* suite (Pape & Schneider, 2004 ▸). The automatic experimental phasing pipeline *CRANK*2 (Skubák & Pannu, 2013 ▸) (using *PRASA* with 2000 trials and a resolution cutoff of 4.2 Å; Skubák, 2018 ▸) provided interpretable electron-density maps and a partial model. About 58% of residues were initially built, so a large proportion had to be rebuilt. Further manual model building in *Coot* was possible after improving the experimental maps by twofold NCS averaging. Refinement was performed with *phenix.refine*.

Five data sets were collected from a single RNAse A crystal at different κ and φ angles. As for SERCA, *XDS*, *XSCALE *(Kabsch, 2010 ▸) and *AIMLESS* were used to process the data sets. *CRANK*2 (using *PRASA* with 2000 trials and a resolution cutoff of 2.4 Å) was able to build an almost complete model that was further corrected and completed in *Coot*. Refinement was performed with *phenix.refine*.

Peak heights in phased anomalous difference maps were computed as follows: firstly, *SHELXC* (Sheldrick, 2010 ▸) was used to calculate anomalous differences based on the reflection files output by *XSCALE* (Kabsch, 2010 ▸). Anomalous differences and the final refined models were fed into *ANODE* (Thorn & Sheldrick, 2011 ▸) to evaluate peak heights.

All data-processing and refinement statistics are shown in Table 1 ▸. The structure factors for MjcD have previously been deposited as PDB entry 5ofr and the high-resolution data set and structure as PDB entry 4pl0. The structures and structure factors for SERCA and RNAse A were deposited in the PDB as entries 6yso and 6yo1, respectively.

## 3. Results   

**Figure 1:**
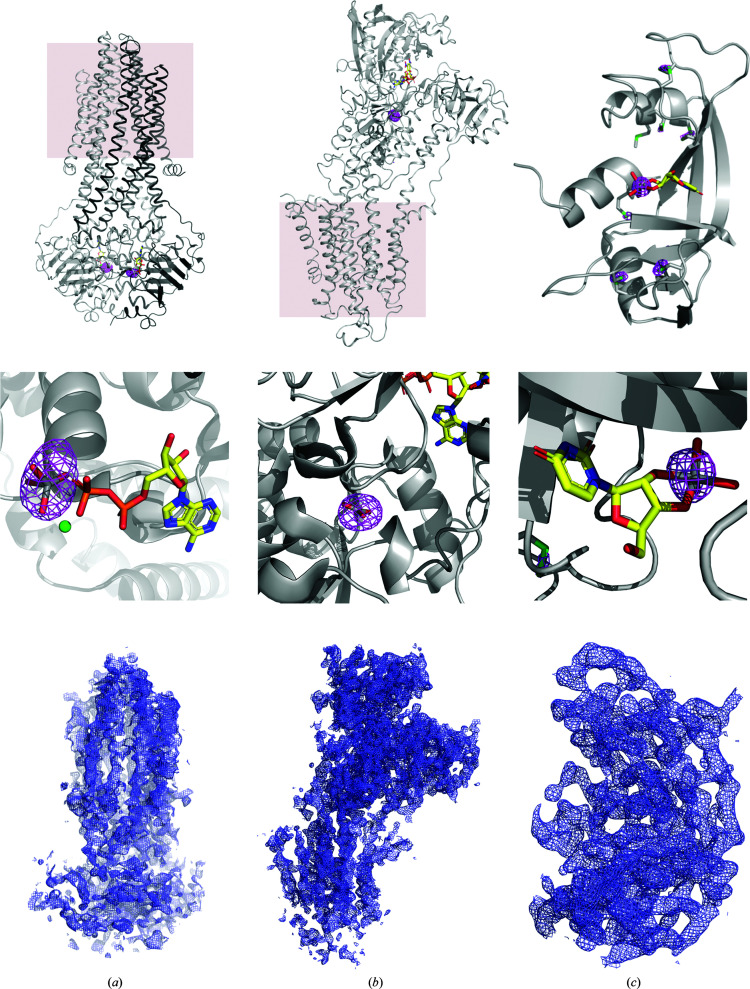
The crystal structures of McjD (*a*), SERCA (*b*) and RNAse A (*c*) were solved by vanadium phasing (top panel). The structures are shown as cartoons and the membrane for McjD and SERCA is depicted in pink. Middle panel: the phased anomalous difference Fourier maps obtained are drawn in magenta and contoured at 7σ for McjD and RNAse A and at 11σ for SERCA. Bottom panel: initial experimental electron-density maps were obtained from the *CRANK*2 pipeline and contoured at 1σ (after density modification and initial model building).

After collecting long-wavelength data for the three proteins, McjD, SERCA and RNAse A, we assessed the strength of the anomalous vanadium signal with the *phenix.anomalous_signal* software (Terwilliger *et al.*, 2016 ▸; Liebschner *et al.*, 2019 ▸). The program predicted a 100% probability of finding the vanadium substructures at the resolution of the data sets (3.4, 3.1 and 1.9 Å, respectively). For comparison, the same experiments with naturally present S atoms as anomalous scatterers gave probabilities of 31%, 26% and 55% for McjD, SERCA and RNAse A, respectively. Nevertheless, finding the vanadium sites does not necessarily warrant successful structure determination; indeed, *phenix.anomalous_signal* predicted a low FOM* for McjD and SERCA (0.3 and 0.2, respectively) and an FOM* of 0.4 for RNAse A, the latter likely to be owing to the higher resolution of the RNAseA data set. Despite the low predicted FOM* values, we successfully determined the crystal structures of McjD, SERCA and RNAse A using vanadium as an anomalous scatterer [Figs. 1 ▸(*a*), 1 ▸(*b*) and 1 ▸(*c*)].

**Figure 2:**
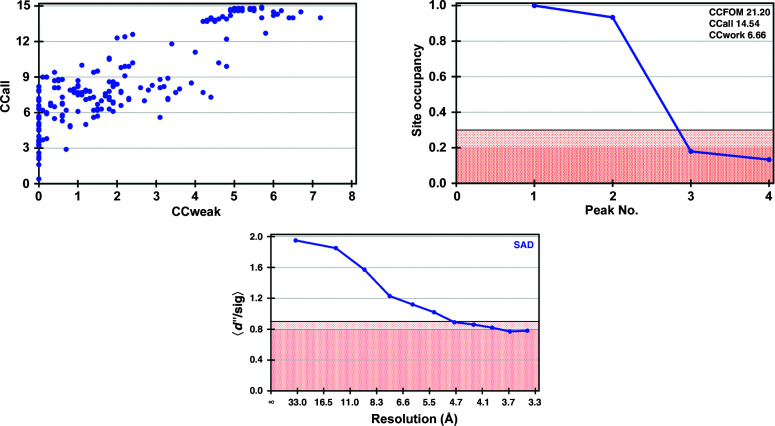
Substructure solutions obtained with *SHELXD* and *HKL*2*MAP* (Pape & Schneider, 2004 ▸; Sheldrick, 2010 ▸) for McjD. Top left: CCall versus CCweak plot (CCall is the correlation coefficient between all normalized structure-factor differences in the measured data and those calculated from a given substructure solution; CCweak is the same but only considers the weak reflections which have not been used for direct methods). Top right: site occupancy versus peak number plot; the pink region corresponds to low site occupancies (doubtful sites). Bottom: *d*′′/sig as function of resolution; the pink region corresponds to the region where the anomalous signal is too weak to be useful for substructure detection.

In our previous work on the McjD–ADP–VO_4_ complex, the presence of vanadium was confirmed by phased anomalous difference Fourier maps (Bountra *et al.*, 2017 ▸; Table 2 ▸). A 360° data set was collected close to the vanadium absorption edge (λ = 2.2604 Å or *E* = 5485 keV) using the inverse-beam method, with an overall multiplicity of only 6.7 (Table 1 ▸). Despite the fact that the McjD–ADP–VO_4_ crystals diffracted poorly to about 3.8 Å resolution and showed severe anisotropy (Supplementary Fig. S2), two vanadium sites (one per monomer) could easily be located (Fig. 2 ▸). The initial experimental electron-density maps were significantly improved when the processed data set was subjected to anisotropy correction to 3.4 Å resolution using *STARANISO* (Tickle *et al.*, 2018 ▸; Table 1 ▸). The modified electron-density maps clearly showed the expected features of McjD [transmembrane and nucleotide-binding domains; Fig. 1 ▸(*c*)]. Owing to the low resolution and anisotropy of the data, model building was not attempted; instead, phase extension was carried out using a higher resolution data set collected at λ = 0.9791 Å with diffraction to 2.7 Å resolution (Table 1 ▸). Automatic and manual model building produced a structure that could be refined with good statistics, as described previously (Choudhury *et al.*, 2014 ▸).

**Figure 3:**
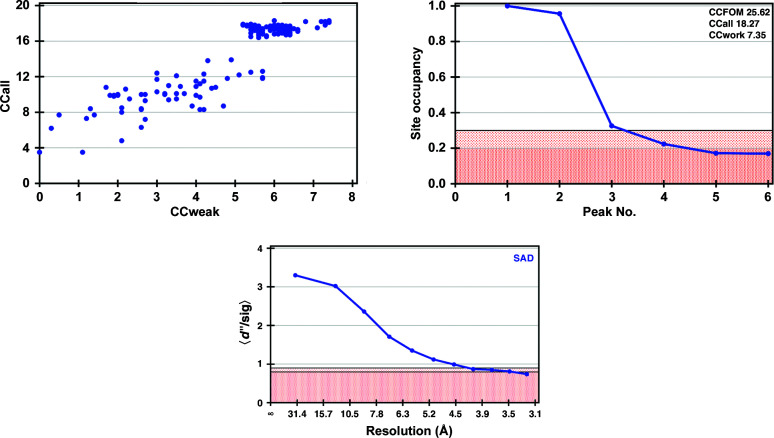
Substructure solutions obtained with *SHELXD* and *HKL*2*MAP* (Pape & Schneider, 2004 ▸; Sheldrick, 2010 ▸) for SERCA. Top left: CCall versus CCweak plot (CCall is the correlation coefficient between all normalized structure-factor differences in the measured data and those calculated from a given substructure solution; CCweak is the same but only considers the weak reflections which have not been used for direct methods). Top right: site occupancy versus peak number plot; the pink region corresponds to low site occupancies (doubtful sites). Bottom: *d*′′/sig as function of resolution; the pink region corresponds to the region where the anomalous signal is too weak to be useful for substructure detection.

SERCA–VO_3_ crystallizes in space group *P*2_1_, with two monomers per asymmetric unit, each of which binds one VO_3_ ion. The crystals diffracted to 3.1 Å resolution, and despite a ratio of one vanadium per 994 protein residues, both of the V atoms could be located (Fig. 3 ▸). The automatic structure-determination pipeline *CRANK*2 (Skubák & Pannu, 2013 ▸) was also able to locate some of the S atoms in addition to the vanadium sites and built an initial model (1690 out of 1988 residues). The sulfur positions were essential to obtain interpretable electron-density maps [Fig. 1 ▸(*b*) and Supplementary Fig. S2], which explains why a higher redundancy of 77 was needed to improve the initial phases. Iterative cycles of manual model building and refinement, further aided by the sulfur peaks in the anomalous difference Fourier maps using the phases from the evolving model, allowed a full structure determination. The final structure had an overall all-atom r.m.s.d. of 0.6 Å compared with the previously reported SERCA–VO_3_ structure determined by molecular replacement.

**Figure 4:**
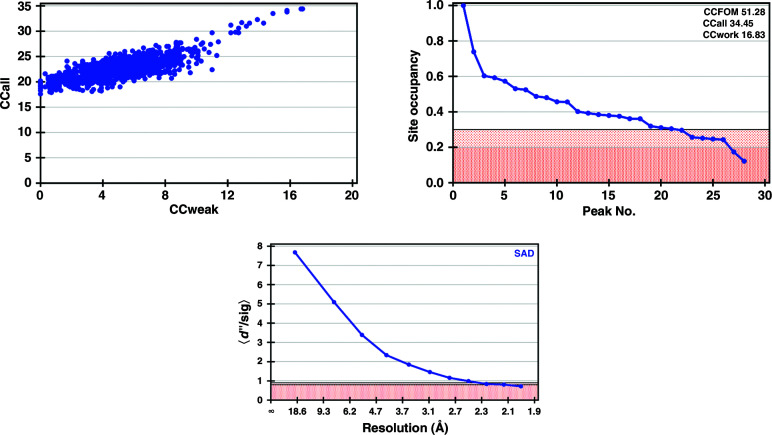
Substructure solutions obtained with *SHELXD* and *HKL*2*MAP* (Pape & Schneider, 2004 ▸; Sheldrick, 2010 ▸) for RNAse A. Top left: CCall versus CCweak plot (CCall is the correlation coefficient between all normalized structure-factor differences in the measured data and those calculated from a given substructure solution; CCweak is the same but only considers the weak reflections which have not been used for direct methods). Top right: site occupancy versus peak number plot; the pink region corresponds to low site occupancies (doubtful sites). Bottom: *d*′′/sig as function of resolution; the pink region corresponds to the region where the anomalous signal is too weak to be useful for substructure detection.

The RNAse A–UV crystals diffracted to 1.9 Å resolution. Five data sets were collected from a single crystal to obtain a multiplicity of 23. Again, the two vanadium sites (two molecules in the asymmetric unit) could easily be found by *SHELXD* (Sheldrick, 2010 ▸; Fig. 4 ▸) and *CRANK*2 (Skubák & Pannu, 2013 ▸), and a complete model was automatically built. Although the two vanadium sites gave the strongest anomalous peaks, the second vanadium had a lower occupancy (Table 2 ▸); this was confirmed by a less well defined electron density for the corresponding uridine.

A second RNAse A–UV crystal was used to compare the effects of higher energies on V-SAD phasing. Two 360° interleaved data sets were collected at λ = 2.2604 Å and λ = 1.7711 Å (*E* = 5485 eV and *E* = 7000 eV, respectively; 45° sweeps were collected alternating between the wavelengths; this ensures that the two data sets are comparable in terms of absorbed X-ray dose). Both data sets were processed in a similar fashion and submitted to *CRANK*2 (Supplementary Table S1).

## 4. Discussion   

Overcoming the challenges of membrane-protein crystallization is the first bottleneck in their successful structure determination. Even if diffracting crystals are obtained, solving a structure often poses further challenges, including poor-homology structures as search models for molecular replacement, the inability to produce labeled recombinant proteins for experimental phasing or non-isomorphism. Previous studies have tried to address this problem by using iodine derivatives or iodinated detergents to overcome the phase problem (Beck *et al.*, 2008 ▸; Dauter *et al.*, 2000 ▸; Melnikov *et al.*, 2017 ▸; Nakane *et al.*, 2016 ▸), but the number of structures determined using this method is still low (24 structures in the PDB). Vanadium complexes are spread over a broad range of enzyme classes in the PDB, but have never been used for phasing purposes. In order to assess the wider applicability of vanadium-based phasing for protein structure determination, we searched the PDB (https://www.rcsb.org) for protein or nucleic acid ligands containing vanadium. Around 150 protein–vanadate or nucleic acid–vanadate complexes were found in total, with 21 different vanadium species listed as ligands. Amongst the entries, eight proteins are membrane proteins, although vanadium was not used in their phasing. The entries encompassed the vanadium ion, different forms of vanadate and vanadate–nucleotide complexes (Supplementary Table S2). Protein–vanadate complexes have been reported in six of the seven EC classes; the ligase family (EC 6) is the only one without a protein–vanadate structural representative in the PDB.

Data sets were collected using a PILATUS 12M detector (Dectris) on beamline I23 at Diamond Light Source (Wagner *et al.*, 2016 ▸). The anomalous signal was measured using a low-dose and high-multiplicity strategy (Weinert *et al.*, 2015 ▸) for the SERCA and RNAse A crystals, whereas only 360° of data were collected from the McjD crystal. The McjD data set was collected using the inverse-beam method, keeping the measurement of Friedel pairs close in time, whereas multiple data sets for SERCA and RNAse A were collected at different κ and φ angles. The latter method has the advantage of increasing the true multiplicity and avoiding any systematic errors owing to the experimental setup (a single reflection is not recorded twice on the same detector pixels; Weinert *et al.*, 2015 ▸).

SAD phasing protocols were chosen over MAD phasing because of the absence of a white line in the vanadate absorption spectrum (Supplementary Fig. S1). In this case MAD is less efficient, as both anomalous and dispersive differences are not as large as they would be for elements that show this feature. Hence, we took the approach of increasing the accuracy of the measured anomalous differences by increasing the data redundancy, in particular as all three of the structures presented here are from crystals in monoclinic space groups. Analysis of the SERCA data sets (Supplementary Table S2) showed that at least four data sets were necessary to obtain an initial model using the automatic phasing pipeline.

We chose a wavelength of λ = 2.2604 Å (*E* = 5485 eV, 20 eV above the theoretical *K* edge) for vanadium phasing. It is worth pointing out that the ratio of the number of anomalous scatterers to the number of residues is low in two of our test cases: 1:580 and 1:994 for McjD and SERCA, respectively. In addition, the crystals of these two proteins had low-symmetry space groups (monoclinic, *C*2 and *P*2_1_) and diffracted to low resolution (3.4 and 3.1 Å; Table 1 ▸). It has to be noted that SAD phasing protocols are generally aided by NCS and a high solvent content. The RNAse A, McjD and SERCA crystals have twofold NCS, and the latter two have relatively high solvent contents of 63% and 66%, respectively, whereas RNAse A has a low solvent content of 44%. The successful phasing of McjD and SERCA shows the potential of vanadate phasing for low-resolution and low-symmetry space groups. Moreover, for both membrane proteins a single crystal was sufficient to determine the structure, avoiding the need to merge data from multiple crystals, which is not possible if the crystals are non-isomorphous. The initial experimental electron-density maps of McjD were interpretable [identification of helices and parts of the nucleotide-binding domain; Fig. 1 ▸(*a*)]; the phases could be extended to a previously published higher resolution data set at 2.7 Å (Choudhury *et al.*, 2014 ▸), facilitating model building.

Since the data sets presented in this paper were collected on a beamline optimized for long wavelengths, the feasibility of performing V-SAD at energies that are more easily reachable by standard beamlines (for example λ = 1.7711 Å, *E* = 7 keV) was also investigated on beamline I23. An interleaved data-collection strategy on one single crystal between λ = 2.2604 Å and λ = 1.7711 Å was used to minimize radiation-damage effects between the two data sets. The interleaved data collection was performed by collecting 360° of data for each wavelength and alternating between the wavelengths every 45° for a single crystal and orientation. The results show, as expected, that the phasing statistics are better at λ = 2.2604 Å but that it is still possible the solve the RNAse A structure at λ = 1.7711 Å (Supplementary Table S1). Taking into account that data collection at λ = 1.7711 Å on a standard beamline will add noise from air scattering, the results can be expected to be worse. Nevertheless, these results open the possibility of V-SAD on standard beamlines, at least for well diffracting crystals.

Another key advantage of using vanadate for phasing comes from the fact that it is a potent inhibitor of a large number of enzymes that catalyze phosphoryl-transfer reactions (Davies & Hol, 2004 ▸). It can bind specifically to the catalytic site of its target enzymes, in contrast to many heavy atoms that bind nonspecifically. Vanadium can furthermore bind in different forms; in fact, 21 different vanadium-containing molecules complexed to proteins or nucleic acids have been deposited in the PDB (Supplementary Table S2). The most common forms are VO_4_ or VO_3_, which adopt a tetrahedral and a trigonal planar geometry, respectively, but vanadate can also bind in its decameric form (Clausen *et al.*, 2016 ▸) and cam bind covalently to serine residues (Holtz *et al.*, 1999 ▸) or the ribose moiety from nucleotides (Ladner *et al.*, 1997 ▸). The latter configuration is seen in the RNAse A–UV complex. As for SERCA and McjD, we decided to examine the potential of V-SAD to phase the structure of RNAse A–UV. The substructure and structure solution were straightforward (Fig. 4 ▸), also considering that the crystal diffracted to beyond 2 Å resolution.

To date, vanadate has been used in protein biochemistry and crystallography to study reaction mechanisms by inhibition, but also to lock proteins in a specific conformation, concomitantly facilitating the progress of crystallization. Our work demonstrates that vanadate can also be used as a phasing reagent for proteins that carry out phosphoryl-transfer reactions. In the PDB, vanadate is found in ∼100 entries, inviting speculation that these structures could have been directly solved by V-SAD. In addition, vanadate forms complexes with enzymes from many enzyme classes, suggesting that it can be exploited as a phasing agent for a broad range of enzymes, including membrane proteins. The fact that its anomalous signal is strong enough to determine large membrane-protein structures that diffract to low resolution, as demonstrated in this study, suggests it could be routinely used for phasing suitable target proteins. Another benefit of collecting diffraction data at the V *K* edge is that the anomalous signal from endogenous S atoms is also significant (*f*′′ = 1.2 electrons), which not only helps the phasing process but can also greatly aid model building at low resolution by locating cysteine and methionine residues from the protein sequence (Supplementary Fig. S3). For higher resolution data, as in our RNAse A example, the height of the sulfur peaks in phased anomalous difference maps is relatively strong (∼8σ; Table 2 ▸). These extra sulfur sites can be found with *SHELXD* (Fig. 4 ▸) and directly contribute to phasing. For SERCA and McjD, only the two vanadium sites could be located with *SHELXD* (Figs. 2 ▸ and 3 ▸), but *CRANK*2 could find additional sulfur sites by analysis of phased anomalous difference maps.

To conclude, our work has provided a novel phasing strategy for both difficult membrane transporters and nucleotide-binding enzymes. Without the need for crystal manipulation by heavy-atom soaking or protein modification for the insertion of selenomethionine or specific amino acids for heavy-atom labeling, V-SAD emerges as a very powerful tool for phasing, since it relies on the inherent chemistry of the proteins of interest. It should be feasible to perform V-SAD on standard beamlines at wavelengths around 1.7711 Å (*E* = 7 keV), especially for well diffracting crystals, but for more difficult projects, such as weakly diffracting crystals, a dedicated beamline for long wavelengths [such as the I23 beamline at Diamond Light Source (Wagner *et al.*, 2016 ▸) or BL-1A at Photon Factory (Liebschner *et al.*, 2016 ▸)] will be more beneficial, if not necessary.

## 5. Related literature   

The following reference is cited in the supporting information for this article: Evans & Pettifer (2001 ▸).

## Supplementary Material

PDB reference: SERCA, 6yso

PDB reference: RNase A, 6yo1

Supplementary Tables and Figures. DOI: 10.1107/S2052252520012312/ro5021sup1.pdf

## Figures and Tables

**Table 1:** Data-collection, phasing and refinement statistics Values in parentheses are for the highest resolution shell.

	McjD, *STARANISO*	McjD, high resolution	SERCA	RNAse A
Data collection				
Beamline	I23, DLS	I02, DLS	I23, DLS	I23, DLS
Space group	*C*2	*P*2_1_2_1_2_1_	*P*2_1_	*C*2
*a*, *b*, *c* (Å)	235.3, 105.1, 117.4	80.8, 107.9, 232.9	131.1, 94.4, 135.1	100.5, 32.7, 72.4
α, β, γ (°)	90, 105.5, 90	90, 90, 90	90, 107.1, 90	90, 90.8, 90
Rotation (°)	360		4320	1800
No. of crystals	1		1	1
Wavelength (Å)	2.2604	0.9791	2.2604	2.2604
Resolution (Å)	57.07–3.40 (3.52–3.40)	48.96–2.70 (2.78–2.70)	129.20–3.13 (3.22–3.13)	41.56–1.90 (1.94–1.90)
No. of unique reflections	22878	56627 (4554)	55958 (4573)	18916 (1194)
Completeness (%)	60.0 (30.3)	99.8 (99.6)	100.0 (100.0)	100.0 (99.6)
Completeness, ellipsoidal (%)	93.5 (94.4)			
Multiplicity	6.7 (6.8)	3.7 (3.8)	77.4 (68.8)	23.6 (18.3)
〈*I*/σ(*I*)〉	14.6 (2.3)	12.1 (1.5)	18 (1.3)	20.6 (1.2)
*R*_merge_ (%)	5.2 (70.9)	5.1 (133.0)	29.9 (598.6)	8.8 (224.5)
*R*_p.i.m._ (%)	2.2 (29.1)		4.8 (1.0)	2.5 (73.1)
CC_1/2_, highest resolution shell	0.88	0.77	0.68	0.57
Anomalous completeness (%)	59.7 (30.1)		99.8 (99.3)	99.0 (95.7)
Anomalous multiplicity	3.4 (3.5)		38.6 (32.5)	11.5 (9.2)
Mid-slope of anomalous normal probability			1.28	1.21
Phasing (*CRANK*2)				
Mean phasing FOM	0.14		0.18	0.19
FOM after density modification	0.28		0.55	0.39
FOM after initial automatic model building			0.81	0.89
No. of residues built after initial automatic model building			1943/2030	247/292
Refinement				
Resolution (Å)		48.96–2.70	64.59–3.13	41.56–1.90
*R*_work_/*R*_free_ (%)		24.8/26.60	19.3/25.7	18.6/21.3
R.m.s.d., bond lengths (Å)		0.01	0.014	0.007
R.m.s.d., angles (°)		1.05	1.15	1.2
Mean *B* factor (Å^2^)		99	121	46
Wilson plot *B* factor (Å^2^)		69	98	36
Ramachandran plot (%)				
Favored		93.1	92.5	97.9
Allowed		6.5	6.9	2.1
Outliers		0.4	0.7	0

**Table 2:** Peak heights in phased anomalous difference maps (σ) computed with *ANODE* (Thorn & Sheldrick, 2011 ▸) for V atoms and S atoms from methionine and cysteine residues

	McjD	SERCA	RNAse A
V (chain *A*)	19.5	33.8	23.1
V (chain *B*)	13.7	30.2	12.1
Averaged V	16.2	31.7	16.8
Averaged methionine SD	2.9	3.2	8.8
Averaged cysteine SG	1.8	3.7	8.4

## References

[bb2] Andersen, J. P., Lassen, K. & Møller, J. V. (1985). *J. Biol. Chem.* **260**, 371–380. 3155517

[bb3] Beck, T., Krasauskas, A., Gruene, T. & Sheldrick, G. M. (2008). *Acta Cryst.* D**64**, 1179–1182. 10.1107/S0907444908030266 19020357

[bb4] Bountra, K., Hagelueken, G., Choudhury, H. G., Corradi, V., El Omari, K., Wagner, A., Mathavan, I., Zirah, S., Yuan Wahlgren, W., Tieleman, D. P., Schiemann, O., Rebuffat, S. & Beis, K. (2017). *EMBO J.* **36**, 3062–3079. 10.15252/embj.201797278 PMC5641919 28864543

[bb5] Bublitz, M., Morth, J. P. & Nissen, P. (2011). *J. Cell Sci.* **124**, 2515–2519. 10.1242/jcs.088716 21768325

[bb6] Carpenter, E. P., Beis, K., Cameron, A. D. & Iwata, S. (2008). *Curr. Opin. Struct. Biol.* **18**, 581–586. 10.1016/j.sbi.2008.07.001 PMC2580798 18674618

[bb7] Choudhury, H. G., Tong, Z., Mathavan, I., Li, Y., Iwata, S., Zirah, S., Rebuffat, S., van Veen, H. W. & Beis, K. (2014). *Proc. Natl Acad. Sci. USA*, **111**, 9145–9150. 10.1073/pnas.1320506111 PMC4078857 24920594

[bb8] Clausen, J. D., Bublitz, M., Arnou, B., Olesen, C., Andersen, J. P., Møller, J. V. & Nissen, P. (2016). *Structure*, **24**, 617–623. 10.1016/j.str.2016.02.018 27050689

[bb9] Cowtan, K. (2006). *Acta Cryst.* D**62**, 1002–1011. 10.1107/S0907444906022116 16929101

[bb10] Dauter, M. & Dauter, Z. (2017). *Methods Mol. Biol.* **1607**, 349–356. 10.1007/978-1-4939-7000-1_14 PMC5557042 28573580

[bb11] Dauter, Z., Dauter, M. & Rajashankar, K. R. (2000). *Acta Cryst.* D**56**, 232–237. 10.1107/s0907444999016352 10666615

[bb12] Davies, D. R. & Hol, W. G. J. (2004). *FEBS Lett.* **577**, 315–321. 10.1016/j.febslet.2004.10.022 15556602

[bb13] Davies, D. R., Mushtaq, A., Interthal, H., Champoux, J. J. & Hol, W. G. J. (2006). *J. Mol. Biol.* **357**, 1202–1210. 10.1016/j.jmb.2006.01.022 16487540

[bb14] Emsley, P., Lohkamp, B., Scott, W. G. & Cowtan, K. (2010). *Acta Cryst.* D**66**, 486–501. 10.1107/S0907444910007493 PMC2852313 20383002

[bb15] Evans, G. & Pettifer, R. F. (2001). *J. Appl. Cryst.* **34**, 82–86.

[bb16] Evans, P. R. & Murshudov, G. N. (2013). *Acta Cryst.* D**69**, 1204–1214. 10.1107/S0907444913000061 PMC3689523 23793146

[bb17] Hendrickson, W. A. (2014). *Q. Rev. Biophys.* **47**, 49–93. 10.1017/S0033583514000018 PMC4128195 24726017

[bb18] Holtz, K. M., Stec, B. & Kantrowitz, E. R. (1999). *J. Biol. Chem.* **274**, 8351–8354. 10.1074/jbc.274.13.8351 10085061

[bb19] Kabsch, W. (2010). *Acta Cryst.* D**66**, 125–132. 10.1107/S0907444909047337 PMC2815665 20124692

[bb20] Ko, Y. H., Bianchet, M., Amzel, L. M. & Pedersen, P. L. (1997). *J. Biol. Chem.* **272**, 18875–18881. 10.1074/jbc.272.30.18875 9228065

[bb21] Ladner, J. E., Wladkowski, B. D., Svensson, L. A., Sjölin, L. & Gilliland, G. L. (1997). *Acta Cryst.* D**53**, 290–301. 10.1107/S090744499601582X 15299932

[bb22] Leonidas, D. D., Shapiro, R., Irons, L. I., Russo, N. & Acharya, K. R. (1997). *Biochemistry*, **36**, 5578–5588. 10.1021/bi9700330 9154942

[bb23] Levina, A., McLeod, A. I. & Lay, P. A. (2014). *Chem. Eur. J.* **20**, 12056–12060. 10.1002/chem.201403993 25088743

[bb1] Liebschner, D., Afonine, P. V., Baker, M. L., Bunkóczi, G., Chen, V. B., Croll, T. I., Hintze, B., Hung, L.-W., Jain, S., McCoy, A. J., Moriarty, N. W., Oeffner, R. D., Poon, B. K., Prisant, M. G., Read, R. J., Richardson, J. S., Richardson, D. C., Sammito, M. D., Sobolev, O. V., Stockwell, D. H., Terwilliger, T. C., Urzhumtsev, A. G., Videau, L. L., Williams, C. J. & Adams, P. D. (2019). *Acta Cryst.* D**75**, 861–877. 10.1107/S2059798319011471 PMC6778852 31588918

[bb24] Liebschner, D., Yamada, Y., Matsugaki, N., Senda, M. & Senda, T. (2016). *Acta Cryst.* D**72**, 728–741. 10.1107/S2059798316005349 27303793

[bb25] McCoy, A. J., Grosse-Kunstleve, R. W., Adams, P. D., Winn, M. D., Storoni, L. C. & Read, R. J. (2007). *J. Appl. Cryst.* **40**, 658–674. 10.1107/S0021889807021206 PMC2483472 19461840

[bb26] Melnikov, I., Polovinkin, V., Kovalev, K., Gushchin, I., Shevtsov, M., Shevchenko, V., Mishin, A., Alekseev, A., Rodriguez-Valera, F., Borshchevskiy, V., Cherezov, V., Leonard, G. A., Gordeliy, V. & Popov, A. (2017). *Sci. Adv.* **3**, e1602952. 10.1126/sciadv.1602952 PMC5429034 28508075

[bb27] Nakane, T., Hanashima, S., Suzuki, M., Saiki, H., Hayashi, T., Kakinouchi, K., Sugiyama, S., Kawatake, S., Matsuoka, S., Matsumori, N., Nango, E., Kobayashi, J., Shimamura, T., Kimura, K., Mori, C., Kunishima, N., Sugahara, M., Takakyu, Y., Inoue, S., Masuda, T., Hosaka, T., Tono, K., Joti, Y., Kameshima, T., Hatsui, T., Yabashi, M., Inoue, T., Nureki, O., Iwata, S., Murata, M. & Mizohata, E. (2016). *Proc. Natl Acad. Sci. USA*, **113**, 13039–13044. 10.1073/pnas.1602531113 PMC5135358 27799539

[bb28] Overington, J. P., Al-Lazikani, B. & Hopkins, A. L. (2006). *Nat. Rev. Drug Discov.* **5**, 993–996. 10.1038/nrd2199 17139284

[bb29] Pape, T. & Schneider, T. R. (2004). *J. Appl. Cryst.* **37**, 843–844.

[bb30] Rose, J. P. & Wang, B.-C. (2016). *Arch. Biochem. Biophys.* **602**, 80–94. 10.1016/j.abb.2016.03.018 27036852

[bb31] Sheldrick, G. M. (2010). *Acta Cryst.* D**66**, 479–485. 10.1107/S0907444909038360 PMC2852312 20383001

[bb32] Skubák, P. (2018). *Acta Cryst.* D**74**, 117–124. 10.1107/S2059798317014462 PMC5947775 29533237

[bb33] Skubák, P. & Pannu, N. S. (2013). *Nat. Commun.* **4**, 2777. 10.1038/ncomms3777 PMC3868232 24231803

[bb34] Terwilliger, T. C., Bunkóczi, G., Hung, L.-W., Zwart, P. H., Smith, J. L., Akey, D. L. & Adams, P. D. (2016). *Acta Cryst.* D**72**, 346–358. 10.1107/S2059798315019269 PMC4784666 26960122

[bb35] Thorn, A. & Sheldrick, G. M. (2011). *J. Appl. Cryst.* **44**, 1285–1287. 10.1107/S0021889811041768 PMC3246834 22477786

[bb36] Tickle, I. J., Flensburg, C., Keller, P., Paciorek, W., Sharff, A., Vonrhein, C. & Bricogne, G. (2018). *STARANISO*. Global Phasing Ltd., Cambridge, UK.

[bb37] Varga, S., Csermely, P. & Martonosi, A. (1985). *Eur. J. Biochem.* **148**, 119–126. 10.1111/j.1432-1033.1985.tb08815.x 3156737

[bb38] Vonrhein, C., Flensburg, C., Keller, P., Sharff, A., Smart, O., Paciorek, W., Womack, T. & Bricogne, G. (2011). *Acta Cryst.* D**67**, 293–302. 10.1107/S0907444911007773 PMC3069744 21460447

[bb39] Wagner, A., Duman, R., Henderson, K. & Mykhaylyk, V. (2016). *Acta Cryst.* D**72**, 430–439. 10.1107/S2059798316001078 PMC4784674 26960130

[bb40] Wang, B.-C. (1985). *Methods Enzymol.* **115**, 90–112. 10.1016/0076-6879(85)15009-3 4079800

[bb41] Weinert, T., Olieric, V., Waltersperger, S., Panepucci, E., Chen, L., Zhang, H., Zhou, D., Rose, J., Ebihara, A., Kuramitsu, S., Li, D., Howe, N., Schnapp, G., Pautsch, A., Bargsten, K., Prota, A. E., Surana, P., Kottur, J., Nair, D. T., Basilico, F., Cecatiello, V., Pasqualato, S., Boland, A., Weichenrieder, O., Wang, B.-C., Steinmetz, M. O., Caffrey, M. & Wang, M. (2015). *Nat. Methods*, **12**, 131–133. 10.1038/nmeth.3211 25506719

[bb42] Winther, A. M., Bublitz, M., Karlsen, J. L., Møller, J. V., Hansen, J. B., Nissen, P. & Buch-Pedersen, M. J. (2013). *Nature*, **495**, 265–269. 10.1038/nature11900 23455424

[bb43] wwPDB Consortium (2019). *Nucleic Acids Res.* **47**, D520–D528. 10.1093/nar/gky949 PMC6324056 30357364

