# Supplementary material for: Experimental phasing with vanadium and application to nucleotide-binding membrane proteins
Source: IUCrJ. 2020 Oct 14;7(Pt 6):1092–101. doi: 10.1107/S2052252520012312 (PMC7642786; doi:10.1107/S2052252520012312)
Supplement: Supplementary file 1 [file m-07-01092-sup1.pdf]

IUCrJ

Volume 7 (2020)

Supporting information for article:

Experimental phasing with vanadium and application to nucleotide-binding membrane proteins

Kamel El Omari, Nada Mohamad, Kiran Bountra, Ramona Duman, Maria Romano, Katja Schlegel, Hok-Sau Kwong, Vitaliy Mykhaylyk, Claus Olesen, Jesper Vuust Moller, Maike Bublitz, Konstantinos Beis and Armin Wagner

Table S1 Comparison between processing and phasing statistics of two RNAse A datasets collected at λ = 2.2604 Å and λ = 1.7711 Å.

Data collection	RNAse A 5485 eV	RNAse A 7000 eV
**Beamline**	DLS-I23	DLS-I23
**Space group**	*C*2	*C*2
**Unit cell (Å, º)**	a=100.4 b=32.9, c=72.5<br>α=γ=90, β=90.4	a=100.4 b=32.9, c=72.5<br>α=γ=90, β=90.4
**Number of degrees, number of crystals**	360°, 1	360°, 1
**Wavelength (Å)**	2.2604	1.7711
**Resolution (Å)**	72.44-1.47 (1.50-1.47)	72.47-1.30 (1.32-1.30)
**Number of unique reflections**	34664 (1007)	54052 (2365)
**Completeness (%)**	86.5 (51.0)	92.3 (81)
**Completeness (%) ellipsoidal**		
**Multiplicity**	4.2 (2.1)	4.6 (4)
**<I/σ(I)>**	21.3 (2.8)	17.8 (1.6)
**Rmerge (%)**	3.4 (18.8)	3.5 (61.7)
**Rpim (%)**	2.4 (18.8)	2.4 (51.4)
**CC1/2 highest resolution shell**	0.88	0.68
**Anomalous completeness**	77.0 (47.7)	84.6 (70.8)
**Anomalous multiplicity**	2.0 (1.1)	2.2 (2.1)
**Mid-Slope of Anom Normal Probability**	1.59	1.19
**Phasing (Crank2)**		
**Substructure CFOM**	53.5	43.5
**Mean phasing FOM**	0.25	0.19
**FOM after density modification**	60.8	51.3
**FOM after initial automatic model building**	0.89	0.92
**Number of residues built after automatic model building/number of fragments**	246/4	239/5
**Rwork/Rfree after model building**	27.4/29.6	28.7/30.8

Table S2 Vanadium-containing ligands deposited in the PDB as of 2019.

Values in parentheses are the number of membrane proteins complexed with vanadium derivatives.

**Ligand identifier**	**Ligand name**	**Number of PDB Entries (membrane proteins)**
**VO4**	TRIOXIDO-OXO-VANADIUM	96 (4)
**VN4**	OXIDO(DIOXO)VANADIUM	12 (1)
**AOV**	ADP ORTHOVANADATE	8 (2)
**AD9**	ADP METAVANADATE	7
**VN3**	TRIOXOVANADIUM	4
**UVC**	URIDINE-2',3'-VANADATE	3
**V5A**	ADENOSINE-5'-VANADATE	3
**V**	VANADIUM ION	3
**AVC**	ADENOSINE-5'-MONOPHOSPHATE-2',3'-VANADATE	2
**V7O**	META VANADATE	2
**V4O**	CYCLO-TETRAMETAVANADATE	2 (1)
**SVA**	SERINE VANADATE	1
**VG1**	ALPHA-D-GLUCOSE-1-PHOSPHATE-6-VANADATE	1
**VO3**	TETRAMETAVANADATE	1
**BVA**	TRIHYDROXY[(N-HYDROXYBENZAMIDATO)OXO]VANADATE	1
**FV1**	DIHYDROXY{[(2R,3S)-3-METHYLOXIRAN-2-YL]PHOSPHONATO-KAPPAO}OXOVANADIUM	1
**AV2**	ADENOSINE-5'-DIPHOSPHATE-2',3'-VANADATE	1
**VA3**	TRIVANADATE	1
**8P8**	C Fe7 S8 V	1
**AIV**	HYDROXY(OXO)BIS(PYRIDINE-2-CARBOXYLATO-KAPPA~2~N,O)VANADIUM(3+)	1
**D6N**	FeV	1

Table S3 Effect of the multiplicity on the structure solution of SERCA

12 datasets (360° each) were processed separately and incrementally merged in XSCALE (Kabsch, 2010). The reflection files were converted to mtz files using Aimless (Evans & Murshudov, 2013) and fed to CRANK2 (Skubak & Pannu, 2013). The table shows that at least 4 datasets are necessary to obtain a starting model.

Number of datasets	Overall SigAno/Anomalous correlation (XSCALE)	Multiplicity (Aimless)	Mean phasing FOM	Density modification FOM	FOM after initial model building	Number of building cycles	% of residues built against sequence/ against built
**1**	0.84/11	6.5	0.151	48.5	0.51	50	4/4
**2**	0.89/14	13	0.144	42.1	0.49	50	2/2
**3**	0.93/17	19.4	0.148	41.5	0.51	50	5/5
**4**	0.97/19	25.8	0.164	54.2	0.81	22	49/56
**5**	1.01/21	32.3	0.173	46.5	0.87	41	67/77
**6**	1.05/23	38.8	0.175	54.1	0.77	15	53/57
**7**	1.08/25	45.2	0.196	56.4	0.8	14	71/77
**8**	1.11/27	51.7	0.192	57.6	0.8	14	61/65
**9**	1.14/30	58.1	0.175	46.9	0.79	16	36/58
**10**	1.16/30	64.6	0.192	57.3	0.85	17	53/59
**11**	1.18/30	71	0.199	57.5	0.85	15	40/63
**12**	1.21/34	77.4	0.182	57.5	0.88	16	58/63

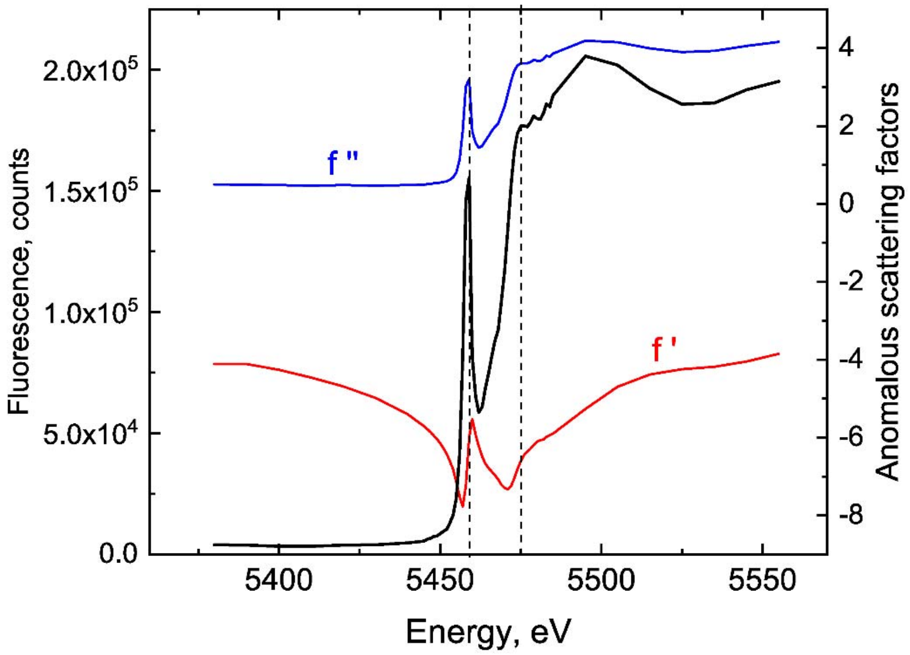

Figure S1 Vanadium K-edge XAS spectra of sodium ortho-vanadate taken on the Diamond I23 beamline (black line). The dashed lines show the pre-edge peak (E = 5459 eV) and the peak (E = 5475 eV with f_0 = 23 e^-, f' = -6.6 e^- and f'' =3.6 e^-) as determined by CHOOCH (Evans & Pettifer, 2001).

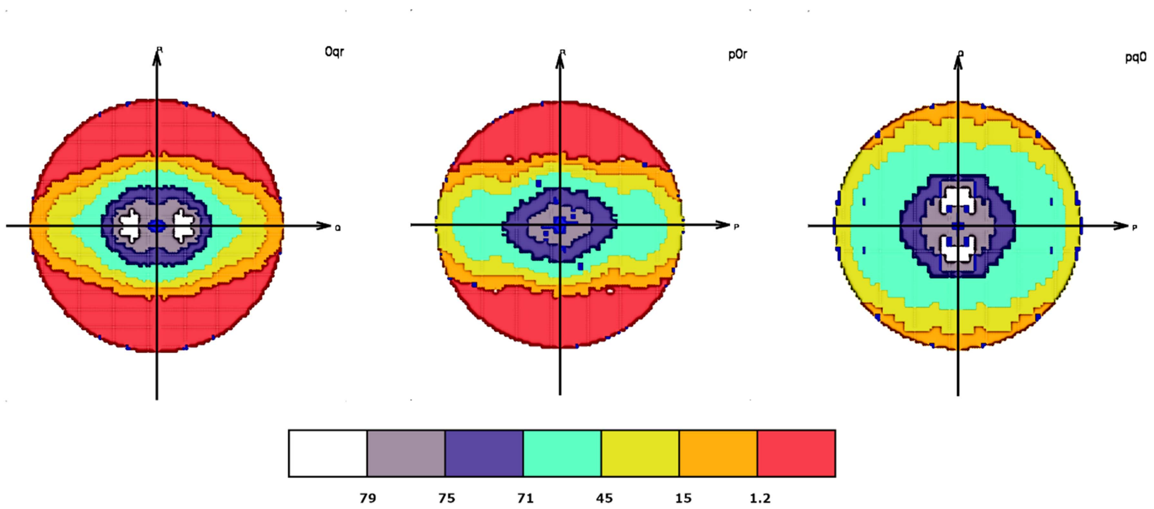

Figure S2 Intensity data in 3D reciprocal space for McjD. The local mean value of $I/\sigma(I)$ for each reciprocal lattice point within the sphere of observation is calculated by STARANISO and colour-coded: the blue points represent unobserved data, the red points represent observed data falling below the 1.2σ threshold for cut-off, and the other colours represent successively higher values of the mean $I/\sigma(I)$ as shown by the colour bar.

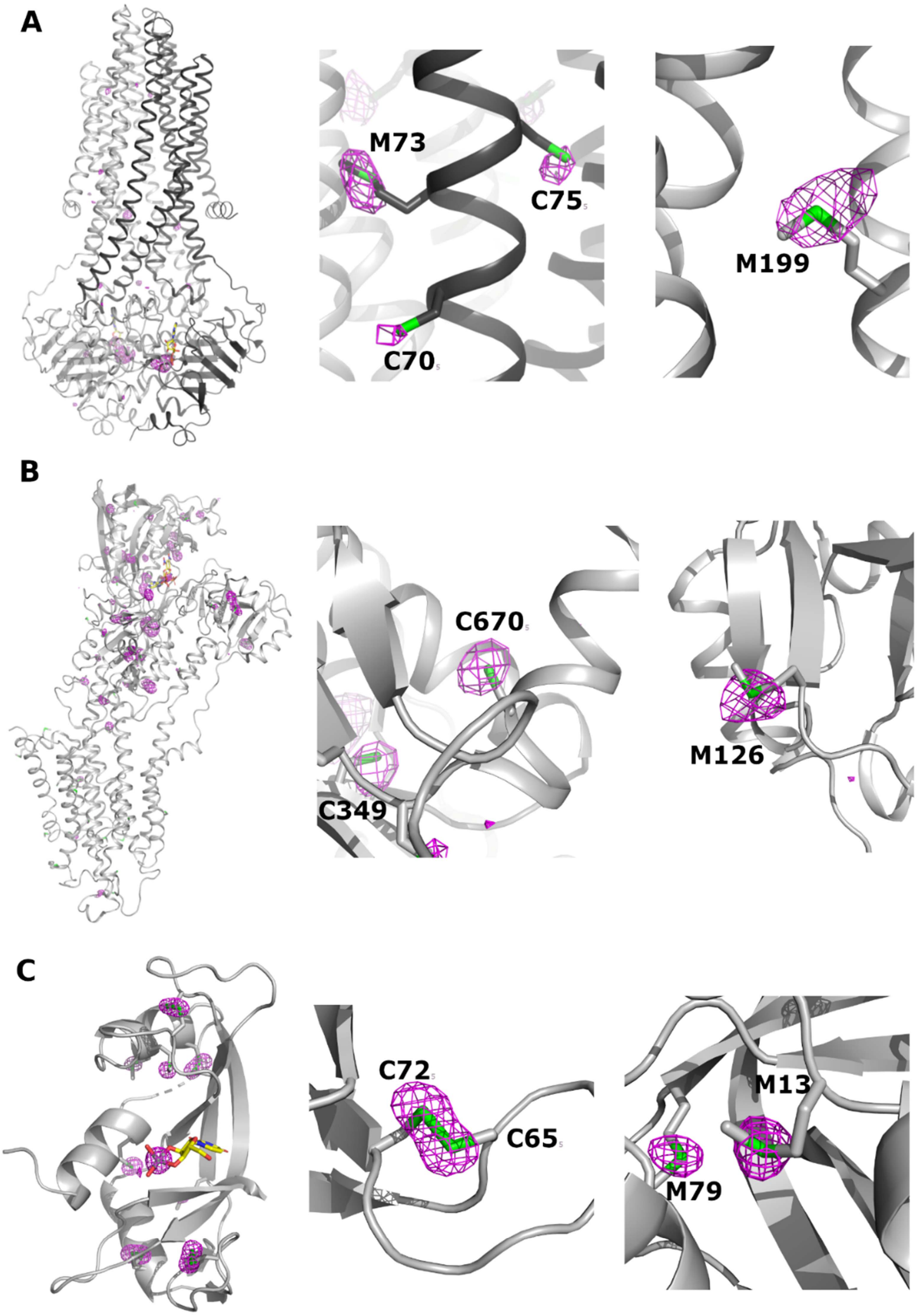

Figure S3 Examples of peaks in phased anomalous difference maps corresponding to sulphur atoms (magenta) calculated with ANODE (Thorn & Sheldrick, 2011). McjD (A), SERCA (B) and RNAse A (C) sulphur peaks are contoured at 3.5, 5 and 3.5 σ respectively.
